# Efficacy of artesunate combined with *Atractylodes lancea* or Prabchompoothaweep remedy extracts as adjunctive therapy for the treatment of cerebral malaria

**DOI:** 10.1186/s12906-023-04150-1

**Published:** 2023-09-20

**Authors:** Walaiporn Plirat, Prapaporn Chaniad, Arisara Phuwajaroanpong, Atthaphon Konyanee, Parnpen Viriyavejakul, Abdi Wira Septama, Chuchard Punsawad

**Affiliations:** 1 Department of Medical Sciences, School of Medicine, Walailak University, Nakhon Si Thammarat, Thailand; 2 Research Center in Tropical Pathobiology, Walailak University, Nakhon Si Thammarat, 80160 Thailand; 3 Faculty of Tropical Medicine, Mahidol University, Bangkok, 10400 Thailand; 4 Research Center for Pharmaceutical Ingredient and Traditional Medicine, National Research and Innovation Agency (BRIN), Cibinong Science Center, Cibinong, West Java 16915 Indonesia

**Keywords:** Prabchompoothaweep remedy, *Atractylodes lancea*, Malaria, Experimental cerebral malaria, Inflammation, Reactive oxidation stress

## Abstract

**Background:**

Cerebral malaria is one of the most serious complications of *Plasmodium* infection and causes behavioral changes. However, current antimalarial drugs have shown poor outcomes. Therefore, new antimalarials with neuroprotective effects are urgently needed. This study aimed to evaluate the effects of selected extracts as monotherapy or adjunctive therapy with artesunate on antimalarial, anti-inflammatory, antioxidant, and neuroprotective properties in experimental cerebral malaria (ECM).

**Methods:**

ECM was induced in male C57BL/6 mice by infection with *Plasmodium berghei* ANKA (*PbA*). Ethanolic extracts of *Atractylodes lancea* (a dose of 400 mg/kg) and Prabchompoothaweep remedy (a dose of 600 mg/kg) were evaluated as monotherapy and adjunctive therapy combined with artesunate at the onset of signs of cerebral malaria and continued for 7 consecutive days. Parasitemia, clinical scores, and body weight were recorded throughout the study. At day 13 post-infection, mouse brains were dissected and processed for the study of the inflammatory response, oxidative stress, blood–brain barrier (BBB) integrity, histopathological changes, and neurocognitive impairments.

**Results:**

Ethanolic extracts of *A. lancea* and Prabchompoothaweep remedy alone improved cerebral malaria outcome in ECM, whereas artesunate combined with extracts of *A. lancea* or Prabchompoothaweep remedy significantly improved the outcome of artesunate and crude extracts alone. Using real-time PCR, *PbA*-infected mice that had received the combination treatment showed significantly reduced gene expression of inflammatory cytokines (TNF-α, IL-1β, IL-6, and IL-10), chemokines (CXCL4 and CXCL10), and adhesion molecules (ICAM-1, VCAM1, and CD36). The *PbA*-infected mice that received the combination treatment showed a significantly decreased malondialdehyde level compared to the untreated group. Similarly, the Evans blue dye assay revealed significantly less dye extravasation in the brains of infected mice administered the combination treatment, indicating improved BBB integrity. Combination treatment improved survival and reduced pathology in the *PbA*-infected group. Additionally, combination treatment resulted in a significantly reduced level of cognitive impairment, which was analyzed using a novel object recognition test.

**Conclusions:**

This study demonstrated that artesunate combined with *A. lancea* or Prabchompoothaweep remedy extracts as adjunctive therapy reduced mortality, neuroinflammation, oxidative stress, BBB integrity protection, and neurocognitive impairment in the ECM.

## Background

Malaria remains a primary medical health concern in many countries worldwide in terms of morbidity and mortality [1]. *Plasmodium falciparum* is the most likely cause of severe symptoms and life-threatening conditions, resulting in an estimated 600,000 deaths annually in Africa [2]. Among the severe complications of *P. falciparum*, cerebral malaria is the deadliest form of malaria, with a mortality rate of up to 30%, especially in African children [3, 4]. According to the World Health Organization, artemisinin and its derivatives are recommended as first-line therapies for severe malaria [5, 6]. Artesunate is preferred in the management of cerebral malaria due to its superior efficacy in comparison to other derivatives [7]. Artesunate eliminates circulating ring-stage parasites, inhibits parasitic maturation, and prevents parasite sequestration into deep organs, including the brain [8]. Although appropriate anti-malarial treatment is available, artesunate monotherapy is insufficient to control the fatality rate due to a lack of specific neuro- and vasculo-protective effects. Furthermore, approximately one-third of cerebral malaria survivors encounter a high risk of neurological impairments [6, 9] such as loss of attention, memory deficits, and learning disabilities [10, 11] despite receiving effective and specific antimalarial treatment [12, 13]. Moreover, the current efficacy of the drug has led to the emergence and spread of artemisinin resistance in Southeast Asia [14]. Therefore, to improve survival and ameliorate the burden of neurological impairment after treatment, new classes of anti-malarial agents and novel strategies for adjuvant therapy are urgently required.

According to botanical evidence, plants and herbs are important sources of molecules for the development of new drugs for many diseases. Natural plants from traditional medicines have provided an opportunity to identify antimalarial molecules, especially quinine and artemisinin, which are also derived from plant extracts [15]. Moreover, plants contain several bioactive compounds and are an important source of pharmacotherapy [16]. Plants have been reported in combination therapy to possess either anti-plasmodial activity or synergy with antimalarial drugs, leading to attenuation of cerebral malaria pathogenesis. Recently, previous studies have demonstrated that a combination of 4-chloro eugenol, a semisynthetic of phytomolecules eugenol, and artesunate showed higher parasitemia suppression and prolonged mean survival time in *P. berghei* infected mice [17]. Oleanane triterpenoids given as adjunctive therapy in combination with artesunate significantly improved survival in ECM through reactive oxygen species scavenging and blood–brain barrier integrity improvement over artesunate alone [18]. In addition, artesunate combined with tetramethylpyrazine, which is a component of the Chuanxiong showed potential adjuvant therapy against ECM by reducing microvascular blockage and improving neurological function [19]. Furthermore, the combination treatment of sinigrin and artesunate demonstrated parasitemia suppression with no recrudescence and enhanced survival in *P. berghei* infected mice [20]. Therefore, plants may be used as adjuvant therapy with additive effects with artesunate or its derivatives in the treatment of cerebral malaria in order to combat its pathogenesis.

Prabchompoothaweep, which is a Thai folk medicine, is used for the treatment of allergic rhinitis and asthma as well as the relief of colds and hay fever, including malaria-like symptom [21]. Prabchompoothaweep is a natural product that produces various secondary metabolites, some of which have antiallergic, anti-inflammation, anti-oxidant, and anti-malarial properties [22]. Piperine, isolated from Prabchompoothaweep remedy, has previously been shown to exert anti-inflammatory effects by inhibiting NO production and Tumor necrosis factor (TNF) release from RAW 264.7 cell lines [23]. A previous study report that the ethanolic extracts of the Prabchompoothaweep remedy displayed a promising anti-plasmodial effects against human malaria parasite *P. falciparum* (IC_50_ = 14.13 ± 0.42 µg/mL) without producing any adverse effects in Vero cells [24]. Furthermore, Prabchompoothaweep remedy shown a potent in vivo anti-plasmodial activity with 60% inhibition in a standard 4-day suppressive model [25]. In addition, *Atractylodes lancea* (*A. lancea*)*,* which is a plant ingredient in Prabchompoothaweep remedy, has been demonstrated to possess promising in vitro anti-plasmodial activity with an IC_50_ value of 7.37 ± 7.72 µg/mL and active in vivo anti-malarial properties with 60% parasitemia suppression [24, 25]. *A. lancea,* belonging to the family Asteraceae (Compositae)*,* has been used in many countries as Chinese, Japanese, and Thai for the treatment of various diseases, including rheumatoid arthritis, gastrointestinal diseases, and influenza [26, 27]. Moreover, *A. lancea* has been linked to anti-cancer, anti-inflammation, anti-microbial properties and activities in the central nervous system [28]. For phase I clinical trial, *A. lancea* extract and its active compound atractylodin significantly suppressed both TNF-α and interleukin-6 (IL-6) expression in Con-A-induced inflammation in human peripheral blood mononuclear cells (PBMCs) [29]. While the pathogenesis of cerebral malaria is complex, it is characterized by marked parasite sequestration, inflammation, oxidative stress and their impact on microvascular function [30]. Interestingly, Prabchompoothaweep remedy and *A. lancea* have shown good activity in anti-malarial properties both in vitro and in vivo and exhibited various medical values, including immunomodulation and anti-oxidant. Therefore, the Prabchompoothaweep remedy and *A. lancea* may serve as an alternative candidate for further studies on ECM model.

In this context, the present study was designed to investigate the anti-plasmodial effect, outcome of cerebral malarial, and neurobehavioral alteration of selected crude extracts from Prabchompoothaweep remedy in the ECM model. Furthermore, the effect of selected crude extracts from Prabchompoothaweep remedy on anti-inflammation and anti-oxidant properties was also determined in the ECM model.

## Methods

### Plant collection

The rhizome*s* of *A. lancea* and twenty-three ingredients of Prabchompoothaweep remedy which consisted of whole plant of *Acanthus ebracteatus*., fruit of *Piper nigrum* L., leaf of *Leonurus sibiricus.,* whole plant of *Kleinhovia hospital* L.*,* flower of *Syzygium aromaticum* (L.) Merr. Et L.M. Perry.*,* whole plant of *Amorphophallus paeoniifolius* (Dennst.) Nicolson.*,* fruit of *Terminalia arjuna* Wight and Arn., fruit of *Terminalia chebula* Retz.*,* roots of *Plumbago indica* L.*,* rhizomes of *Zingiber officinale* Roscoe, fruit of *Lepidium sativum* L.*,* fruit of *Anethum graveolens* L.*,* fruit of *Foeniculum vulgare* Miller subsp. Var. Vulgare*.,* seed of *Nigella sativa* L., rhizome of *Angelica dahurica* Benth.*,* rhizome of *Atractylodes lancea* (Thung.) DC.*,* fruit of *Ardisia elliptica* Thunb.*,* fruit of *Enhalus acoroides* Zolls*.,* flower of *Piper chaba* Hunt., seed of *Myristica fragrans* Houtt*.*, aril of *Myristica fragrans* Houtt., fruit of *Amomum testaceum* Ridl., and leaf of *Cinnamomum camphora* (L.) J. Presl. were obtained from a Thai traditional medicine drug store in Nakhon Si Thammarat Province, Thailand. Permissions for the collection of plant materials were granted in accordance with the guidelines and regulations of Thailand’s Plant Varieties Protection, Department of Agriculture, Ministry of Agriculture and Cooperatives. Botanical authentication and identification were previously established by a botanist at the School of Pharmacy, Walailak University. The voucher specimen numbers were deposited in the School of Medicine, Walailak University as reported in a previous study [24].

### Preparation of *Atractylodes lancea* and prabchompoothaweep extracts

Initially, the rhizomes of *A. lancea* and Prabchompoothaweep remedy were cleaned and desiccated to remove humidity, and then ground into a coarse powder using a traditional herbal pulverizer (Jincheng, model; SF, China). The powdered material of *A. lancea* (60 g) and Prabchompoothaweep remedy (60 g) was immersed in a flask containing approximately 0.6 L of 95% ethanol for 72 h at 25 ^o^ C (1:10 (*w/v*) ratio). The fermented solution was permeated with gauze and Whatman No. 1 filter paper. The residuals were re-extracted twice by adding an equal volume of fresh 95% ethanol for 72 h. The combined solutions were concentrated and evaporated under reduced pressure using a rotary evaporator (Buchi® rotary evaporator; Model R-210, Shanghai, China). The residues were heated to dryness in a water bath at 60° C; the residues were slightly heated to dry. The dried extracts of *A. lancea* and Prabchompoothaweep remedy were then stored in a screw-top container and refrigerated at 4 ^o^ C until use. The crude extract should be used within 2 months for its stable bioactivity [31, 32]. Each crude extract was dissolved in 7% Tween 80 and 3% ethanol in distilled water to provide a working solution for animal experiments.

### Animals and malaria parasite

Healthy male C57BL/6 mice, aged 6–8 weeks and weighing 25–30 g was obtained from Nomura Siam International Co. Ltd., Bangkok, Thailand. The animals were housed in groups of five per cage for 7 day under pathogen-free conditions in standard laboratory conditions (22 ± 3° C, relative humidity of approximately 50–60%, and under 12–12 h light–dark cycles). A standard laboratory diet and clean water were provided ad libitum. The waste from the cages was disposed of and cleaned daily by animal care staff. The mice were handled in accordance with the international guidelines for the ethical use of laboratory animals. The rodent malaria parasite *Plasmodium berghei* ANKA (*PbA*) strains were kindly provided by Thomas F. McCutchan and obtained from the Biodefense and Emerging Infections Research Resources Repository (BEI Resources), the National Institute of Allergy and Infectious Diseases, and the National Institute of Health. Mouse donors received an intraperitoneal injection of *PbA*-infected red blood cells suspended in 0.1 mL of phosphate-buffered saline. Blood was drawn from the heart by cardiac puncture and collected in a heparinized tube for injection into experimental mice when the parasitemia levels of mouse donors reached 20–30%.

### Animal grouping and dosing

For the ECM test, male C57BL/6 mice were randomly divided into seven groups of 10 mice each. Group 1 (uninfected control group) received a mixture of 7% Tween 80 and 3% ethanol in distilled water. Group 2 (negative control group) was infected mice received a mixture of 7% Tween 80 and 3% ethanol in distilled water. Group 3 (positive control) was infected mice received 6 mg/kg body weight artesunate (Art). Group 4 was infected mice received 400 mg/kg body weight of *A. lancea* (AL) crude extract. Group 5 was infected mice received 600 mg/kg body weight of Prabchompoothaweep (PT) crude extract. Group 6 (Art-AL combination) was infected mice received artesunate (6 mg/kg body weight) and 400 mg/kg body weight of *A. lancea* crude extract. Group 7 (Art-PT combination) was infected mice received 6 mg/kg body weight of artesunate and 600 mg/kg body weight of Prabchompoothaweep remedy crude extract. The doses used in the experiment were selected based on the effects of in vivo antimalarial activity and acute oral toxicity of the crude extracts.

### Mouse model of experimental cerebral malaria

Male C57BL/6 mice were intraperitoneally injected with 0.1 mL of infected blood containing 1 × 10^7^ *PbA* parasite red blood cells on day 0 (D0). At 6 days post-infection, the mice were randomly divided into seven groups, as described above. The crude extract was administered to the mice in each group once a day for 7 consecutive days. Treatment was performed by oral gavage to imitate the traditional route of administration. The development of the experimental model was assessed by evaluating the parasitemia level, clinical symptoms, and body weight. The percentage of weight loss compared with D0 was calculated to determine the ability of the treatments to prevent weight loss due to infection. The 10 parameters of clinical symptoms were evaluated every 2 days, starting from D4 to D12, to assess a rapid murine cerebral behavioral score (RMCBS), as described by Carroll et al. [33] to monitor the onset of neurological signs. The clinical score was evaluated in an experiment consisting of gait pattern, balance, exploratory behavior, body position, muscle strength, grooming, touch escape reflex, pinna reflex, toe pinch reflex, and aggressiveness. Each parameter was scored on a scale of 0–2, with 0 indicating a severe neurological deficit and 2 indicating the least severe neurological deficit. All observers were blinded to the group assessments. To minimize potential suffering, the animals were euthanized and excluded from the experiment when signs of pain were noticed. Parasitemia was measured using a thin blood smear film from the vascular tail vein from D3 to D12. Thin blood smears were fixed and stained using 10% Giemsa solution (Biotech Reagent Company Limited, Bangkok, Thailand). Parasitemia was determined using a light microscope (Olympus, model: CX-31, Tokyo, Japan) with a 100X objective lens. The percentage of blood parasites was calculated using 300 red blood cells from five different fields. The average percentage of parasitemia was calculated using the following formula:

$$\%\text{parasitemia}=\frac{\text{number of parasitized red blood cells}}{\text{total number of red blood cells counted}}\times{100}$$

The percentage of parasitemia suppression was measured using the following formula:

$$\%\mathrm{suppresion}=\frac{\left[\mathrm{A}-\mathrm{B}\right]}{\mathrm{A}}\times 100$$

where A is the average percentage of parasitemia in the negative control group and B is the average percentage of parasitemia in the test group.

On day 13, the mice were euthanized by anesthesia containing 2% isoflurane (Piramal Pharma, PA, USA) by inhalation via rodent anesthesia machines and then euthanized by cardiac puncture. After euthanization, brain tissue was harvested for studies of histopathological changes, malondialdehyde levels, and gene expression levels.

### Rapid murine coma and behavioral assessment

At day 4 post-infection, behavioral clinical signs and disease severity were assessed using the grading quantitative RMCBS protocol. This method was used to evaluate the clinical manifestations of murine cerebral malaria and the degree of morbidity associated with neurodegenerative disorders. The protocol consists of 10 parameters: coordination (gait and balance), exploration behavior, strength and tone (body position and limb strength), reflex and self-preservation (toe reflex, pinna reflex, touch reflex, and aggressiveness), and hygiene-related behavior. Murine cerebral malaria was defined as the manifestation of one or more of the following symptoms of neuropathological involvement: abnormal gait, muscle weakness, poor righting reflex, convulsions, rolling over, and unconsciousness or coma. The clinical manifestations of murine cerebral malaria were assessed and used to score the severity of the disease and guide the initiation of anti-malarial treatment.

In brief, the protocol took approximately 3 min to evaluate all the parameters. For the first 90 s, the animals were recorded on video to evaluate their gait pattern, motor performance, body position, reflex and self-preservation, grooming, and aggressiveness. During the next 90 s, the animals were observed for limb strength and balance. Each item was scored from 0, which is correlated with severe neurological impairment, to 2, which is correlated with the lowest neurological deficit. The total score of the parameters was calculated as the sum of each domain on the day of analysis.

### Gene expression evaluation of inflammatory cytokine, chemokine, and adhesion molecule markers in brain tissues using qRT-PCR

On day 13 after infection (D13), brains were collected to isolate RNA using GENEzol™ reagent (Catalog no. GZR100, Geneaid, Taiwan), according to the manufacturer’s instructions. Total RNA was extracted using a NanoDrop1000. Total RNA (2 µg of total RNA was added to DEPC-treated water (BIO BASIC CANADA INC., Ontario, Canada) to eliminate residual genomic DNA. Next, the RNA sample was reverse-transcribed into cDNA using iScript™ Reverse Transcription Supermix for qRT-PCR (Catalog no. 1708840, Bio-Rad, USA). The qRT-PCR was performed on a QuantStudio™ 3 Real-Time PCR System using 5X HOT FIREPol® EvaGreen® qPCR Mix Plus (ROX) (Catalog no. 08–24-00001, Solis BioDyne, Estonia). The specified thermocycling conditions were used to perform PCR as follows: an initial denaturation with one cycle at 95 ^o^ C for 15 min, 34 cycles at 95 ^o^ C for 30 s, 62 ^o^ C for 30 s, and followed by a final extension at 72 ^o^ C for 15 min. Each condition was tested in at least two independent experiments. The expression level of each gene was normalized to that of GAPDH, and relative gene expression was calculated using the ΔΔCt method. The primers used are listed in Table 1.

**Table 1:** List of murine primers used in quantitative qRT-PCR experiments [34–38]

Gene	Primer	Sequence 5′-3′	Product size (bp)
TNF-α	Forward	5′-CTCCCTTTGCAGAACTCAGG-3′	211
	Reward	5′-AGCCCCCAGTCTGTATCCTT-3′	
IL-1β	Forward	5′-CTAAAGTATGGGCTGGACTG-3′	168
	Reward	5′-GGCTCTCTTTGAACAGAATG-3′	
IL-6	Forward	5′-AATGATGGATGCTACCAAAC-3′	212
	Reward	5′-TAGCCACTCCTTCTGTGACT-3′	
IL-10	Forward	5′-TTTAAGGGTTACTTGGGTTGCC-3′	146
	Reward	5′-CGCATCCTGAGGGTCTTCA-3′	
CXCL4	Forward	5′-CAGTCCTGAGCTGCTGCTTCT-3′	153
	Reward	5′-TCCAGGCTGGTGATGTGCTTA-3′	
CXCL10	Forward	5′-GCCGTCATTTTCTGCCTCAT-3′	127
	Reward	5′-GCTTCCCTATGGCCCTCATT-3′	
ICAM-1	Forward	5′-AGCTTGCACGACCCTTCTAA-3′	159
	Reward	5′-AGCACCTCCCCACCTACTTT-3′	
VCAM-1	Forward	5′-GTTTGCAGTCTCTCAAGCTTTT-3′	66
	Reward	5′-CCGATTTGAGCAATCGTTT-3′	
CD36	Forward	5′-GAGCAACTGGTGGATGGTTT-3′	206
	Reward	5′-GCAGAATCAAGGGAGAGCAC-3′	
GAPDH	Forward	5′-ACACATTGGGGGTAGGAACA-3′	222
	Reward	5′-AACTTTGGCATTGTGGAAGG-3′	

### Oxidative stress assay

The level of malondialdehyde (MDA) was evaluated using a commercial kit, according to the manufacturer’s instructions (MAK085, Sigma-Aldrich, St. Louis, USA). Briefly, the brain (50 mg) was collected and lysed with 0.3 mL of MDA lysis buffer containing 3 µL butylated hydroxytoluene (100X BHT) on ice. Then, the solution was precipitated at 10,000 × g for 10 min to remove insoluble substances. The supernatant was then transferred to and stored in a microcentrifuge tube. Approximately 0.1 M MDA standard solution was prepared by mixing 10 µL of 4.17 M MDA standard solution with 0.407 mL of ultrapure water. Next, 20 µL of the 0.1 M MDA standard solution was mixed with 0.98 mL of ultrapure water to prepare a 2 mM MDA standard. To prepare concentrations starting at 0 (blank), 0.4, 0.8, 1.2, 1.6, and 2.0 nM standards, volumes of 0, 2, 4, 6, 8, and 10 µL of the 2 mM MDA standard solution were pipetted into microcentrifuge tubes, and ultrapure water was added to each tube to obtain a final volume of 0.2 mL. The sample or standard solution was mixed with 600 µL of thiobarbituric acid solution and incubated at 95 °C for 60 min. After incubation, the tube was transferred to an ice bath and cooled for 10 min, and 200 µL of the solution was aspirated from the microcentrifuge tube and dropped into a 96-well plate. The samples in each condition were analyzed in triplicate. The optical density was measured at 532 nm, and the MDA level was calculated using the following formula:

$$\text{MDA concentration} = \frac{\text{The amount of MDA in an unknown sample from the standard curve}}{\text{The sample volume (mL) or the amount (mg) added to the wells}} \times \text{The sample dilution factor}$$

### Brain histopathological examination

Histopathological examination was performed using a standard laboratory procedure according to previous reports [39, 40]. On day 13, the mice were anesthetized with 2% isoflurane (Piramal Pharma, PA, USA) by inhalation via rodent anesthesia machines and then euthanized by cardiac puncture. The brain was dissected and routinely processed for fixation in 10% (v/v) formalin at room temperature, dehydrated with a series of alcohol concentrations (50%, 70%, 90%, 100% of ethanol), and cleared with xylene solution. Brains were then molded using paraffin wax. The paraffin-embedded tissue was then dissected to a thickness of 5 µm using a microtome and stained with hematoxylin and eosin. Brain tissues were examined in the cortical-medullary area under a light microscope by two independent observers who were blinded to the treatment group.

### Evan blue dye perfusion for brain vascular permeability

Evans blue dye extravasation was examined to evaluate brain vascular leakage as previously described by Kim et al. [41]. Briefly, on day 13 post-infection, mice from all the experimental groups were anesthetized using 2% isoflurane (Piramal Pharma, PA, USA) by inhalation via a rodent anesthesia machine. Next, a 2% Evans Blue dye (catalog no. E2129, Sigma-Aldrich, St. Louis, USA) was diluted in a sterile saline solution and 0.2 mL of the solution was transcardially injected for approximately 5 min. After Evans blue dye injection, animals were perfused through the left ventricle of the heart with 0.9% saline solution. The brains were collected, weighed, photographed, and immediately frozen at -80 ^o^ C for later processing. The brains were placed in dimethyl formamide (catalog no. D4551, Sigma-Aldrich, St. Louis, USA) for 48 h at room temperature (in the dark) to extract the Evans blue dye from the brain tissue. After 48 h, 0.2 mL of this supernatant was quantified in duplicate using spectrophotometry, and the absorbance was measured at 620 nm. For quantification, the results were normalized to the brain dry weight.

### Novel object behavioral test

The novel object behavioral test was used to evaluate the long-term working memory of mice and was performed according to a previous protocol [42]. The novel object behavioral test was performed on days 12 and 13 post-infection, and the protocol consisted of three parts: habituation, training, and testing sessions (Fig. 6A). In brief, the animals were acclimatized and freely explored in an empty testing chamber (open field 50 cm × 50 cm × 50 cm square) for 5 min in a habituation session. Subsequently, a training session was performed with two identical objects (objects A and A’) that were placed in the two opposite connections (10 cm from the walls) of the chamber for 5 min. The testing session was conducted 24 h after the training session. Mice were placed in a chamber for 5 min with one of the same objects (A) and another novel object (B). The objects were distinct in shape and color. The animal’s nose toward the object (at a distance of 2 cm) and rearing on the object indicated that it was exploring. The chamber and objects were cleaned with 70% alcohol after trials to prevent olfactory cues. Behavioral tests were recorded using a video camera mounted above the testing chamber, and the results were analyzed by an observer who was not aware of the group conditions. According to Bevins and Besheer [43], exploratory preference is defined as the percentage of the total time spent with novel objects. The percentage of discrimination is calculated using the following formula:

$$\%\text{total exploration time anime spent}=\frac{\text{TB}}{\text{TA}+\text{TB}}$$

TA: time spent exploring the familiar object.

TB: time spent exploring the novel object.

### Statistical analysis

Statistical analyses were performed using SPSS statistical software version 23 (IBM, Armonk, NY, USA). Quantitative results are expressed as the mean ± standard error of the mean (mean ± SEMs). All variables of each parameter were tested for normality using the Kolmogorov–Smirnov test. Differences in the mean parameters between the groups, such as the percentage of parasitemia, percentage of suppression, RMCBS score, body weight, mRNA quantification, Evans blue dye quantification, and behavioral examination, were compared using one-way analysis of variance followed by Bonferroni’s post-test. For all tests, the statistical significance level was set at *p* < 0.05.

## Results

### Neurological symptoms and antimalarial activity in ECM

**Fig. 1:**
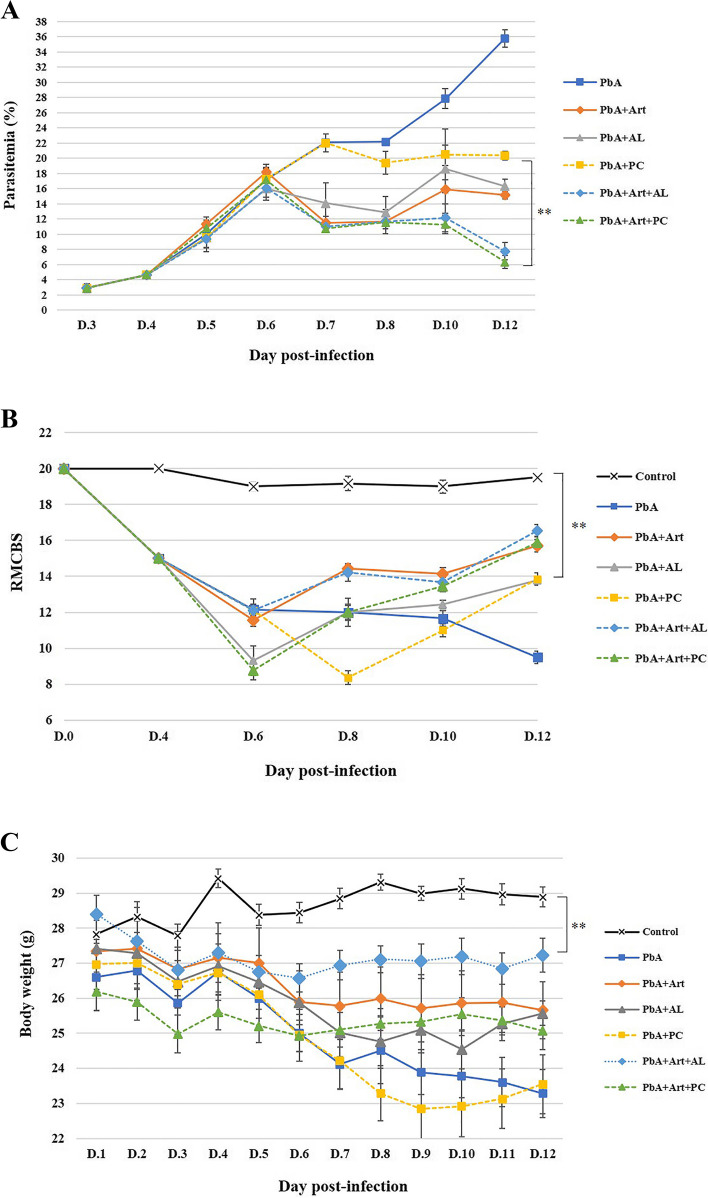
Evaluation of selected crude extracts from Prabchompoothaweep remedy in experimental cerebral malaria (ECM) model. C57BL/6 mice are inoculated with 1 × 10^7^ *P. berghei* ANKA and treated with artesunate, crude extract from Prabchompoothaweep remedy, or 7% Tween 80 from day 6 to day 12 post-infection. **A** The parasitemia levels are evaluated through thin blood smear film from the vascular tail vein beginning on days 3, 4, 5, 6, 7, 8, 10, and 12. Parasitemia levels are calculated from the number of parasitized red blood cells in at least 300 red blood cells. **B** Clinical score of mice at days 0, 4, 6, 8, 10, and 12 post-infection. **C** Body weight variation of mice during infection. ECM, experimental cerebral malaria; RMCBS, Rapid murine coma and behavior score; *PbA*, *Plasmodium berghei* ANKA,;Art, artesunate; AL, *A. lancea*; PT, Prabchompoothaweep remedy. Results are shown as mean ± standard error of the mean (SEM) (*n* = 5 per group). * *p* < 0.05 vs. *PbA*, ** p < 0.001 vs. *PbA*

On the day 6 post-infection, approximately 90% of C57BL/6 mice infected with *PbA* developed marked neurological symptoms, including inactive physical movement, weakness, an abnormal gait pattern, an abnormal righting reflex, and a lack of aggression. Thirteen days post-infection, the *PbA*-infected group showed severe CM signs, including ataxia gait, motor muscle weakness, rolling, seizures, and convulsions. In contrast, the mice that received adjunctive therapy with artesunate combined with AL or PT did not develop neurological signs of CM and survived until the end of the study (Fig. 1A). All treatment groups were checked for parasitemia from day 3 post-infection until the end of the study. The *PbA*-infected group developed a progressive increase in parasitemia between days 3 and 12 post-infection, indicating disease progression. At day 12 post-infection, regarding monotherapy testing, *PbA-*infected group administered with the extracts of *A. lancea* (400 mg/kg body weight) and Prabchompoothaweep remedy (600 mg/kg body weight) showed significant suppression of parasitemia with 54.41 ± 2.63 and 43.13 ± 1.72, respectively, compared to the *PbA*-infected-untreated group (*p* < 0.05) (Table 2). Furthermore, for adjunctive therapy testing, the average parasitemia levels in both Art-AL combination and Art-PT combination groups were significantly lower from day 7 to day 12 post-infection (78.27 ± 3.15 and 82.27 ± 2.38, respectively) compared to the *PbA*-infected-untreated group (*p* < 0.05) (Table 2).

**Table 2:** Effects of selected crude extract from Prabchompoothaweep remedy on parasite level (day 6 and day 12 post-infection) and parasite suppression in experimental cerebral malaria (ECM) mice model at day 12 post-infection

Group	Dose (mg/kg)	% Parasitemia		% Suppression (Day 12)
		**Day 6**	**Day 12**	
*P. berghei* (PbA)	-	17.18 ± 1.49	35.80 ± 1.13 ^b, c, d, e, f^	-
PbA + Artesunate (Art)	6	18.17 ± 1.01	15.14 ± 0.51 ^a, d, e, f^	57.70 ± 1.43 ^d, e, f^
PbA + *A. lancea* (AL)	400	16.02 ± 1.54	16.32 ± 0.94 ^a, d, e, f^	54.41 ± 2.63 ^d, e, f^
PbA + Prabchompoothaweep (PT)	600	17.28 ± 1.48	20.35 ± 0.61^a, b, c, e, f^	43.13 ± 1.72 ^b, c, e, f^
PbA + Art + AL	Art 6 + AL 400	16.08 ± 1.22	7.78 ± 1.13 ^a, b, c, d^	78.27 ± 3.15 ^b, c, d^
PbA + Art + PT	Art 6 + PT 600	17.16 ± 0.49	6.34 ± 0.85 ^a, b, c, d^	82.27 ± 2.38 ^b, c, d^

Results are shown as mean ± standard error of the mean (SEM) (*n* = 5 per group), *p* < 0.05. ^a^ compared to *PbA*-infected mice, ^b^ compared to artesunate, ^c^ compared to 400 mg/kg of *A. lancea*, ^d^ compared to 600 mg/kg of Prabchompoothaweep remedy, ^e^ compared to artesunate and *A. lancea* combination, and ^f^ compared to artesunate and Prabchompoothaweep remedy combination

During active infection, the *PbA-*infected-untreated and treated groups receiving the ethanolic extract of Prabchompoothaweep remedy showed a progressive reduction in body weight (estimated- 14% or greater). On day 12 post-infection, the artesunate (- 6.54%) and treated groups that received the ethanolic crude extract of *A. lancea* (- 7.19%) had lower body weight changes than the *PbA-*infected-untreated group. Furthermore, the Art-AL (- 4.26%) and the Art-PT combination groups (- 4.46%) showed significantly lower body weight changes than the *PbA-*infected-untreated group (*p* < *0.05*) (Fig. 1C).

### Quantitative assessment of cerebral malaria using the RMCBS

A quantitative assessment of neurological impairments caused by the manifestation of CM was performed using the RMCBS score at days 4, 6, 8, 10, and 12 post-infection (Fig. 1B). On day 6 post-infection, *PbA*-infected mice exhibited neurological signs of CM, as indicated by the reduced RMCBS score*.* At day 12 post-infection, *PbA*-infected untreated mice had the lowest score for symptoms, such as inactive exploration behavior, ruffled fur, abnormal reflexes, muscle weakness, poor self-preservation, and convulsions*.* Interestingly, the RMCBS scores of the *PbA*-infected group receiving ethanolic crude extracts of *A. lancea* and Prabchompoothaweep remedy were significantly higher than those of the *PbA*-infected-untreated group (*p* < 0.05) during the same period. Moreover, *PbA*-infected groups that received artesunate, Art-AL, and Art-PT combinations presented higher RMCBS scores than the *PbA*-infected-untreated group (*p* < 0.05).

### Gene expression of inflammatory cytokines, chemokines, and adhesion molecules in the brain tissues

**Fig. 2:**
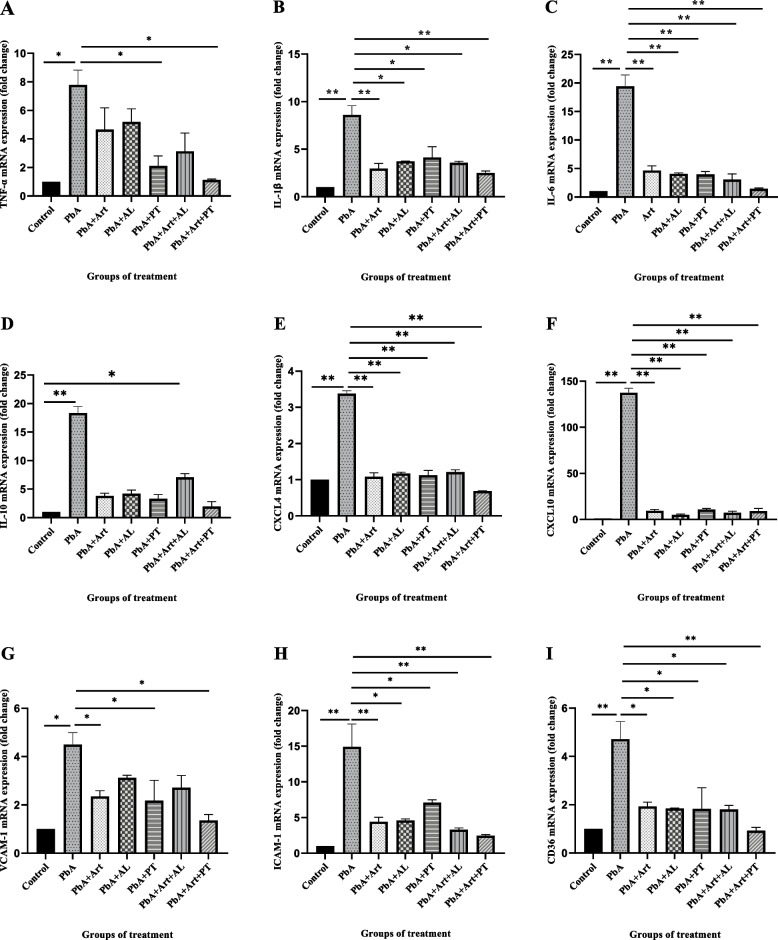
Comparison of the effect of selected crude extracts from Prabchompoothaweep remedy on inflammatory cytokine, chemokine, and adhesion molecule. *PbA*-infected mice are administered *A. lancea*, Prabchompoothaweep remedy, Art-AL combination, and Art-PT combination, and qRT-PCR was performed on the brain at day 13 post-infection. Gene expression levels of inflammatory cytokine (**A**: TNF-α; **B**: IL-1β; **C**: IL-6; and **D**: IL-10), chemokine (**E**: CXCL4 and **F**: CXCL10), and adhesion molecule (**G**: ICAM-1, **H**: VCAM-1, and **I**: CD36) were measured in the brain. Results are demonstrated as mRNA expression of the infected-untreated group, artesunate, *A. lancea* crude extract, Prabchompoothaweep remedy crude extract, Art-AL combination group, or Art-PT combination group, relative to the control group. The relative levels of gene expression are normalized to those of GAPDH in each sample, and the gene mRNA relative expressions are calculated using the threshold cycle (Ct) method. *PbA, Plasmodium berghei* ANKA; Art, artesunate; AL, *A. lancea*; PT, Prabchompoothaweep remedy. Results are shown as mean ± standard error of the mean (SEM) (*n* = 5 per group) and are representative of at least two independent experiments. * *p* < 0.05 versus *PbA*, ** *p* < 0.001 versus *PbA*

On day 13 post-infection, the gene expression of inflammatory cytokines, chemokines, and adhesion molecules in brain tissues was measured using qRT-PCR. The results showed that the gene expression of TNF-α (Fig. 2A), IL-1β (Fig. 2B), and IL-6 (Fig. 2C) in *PbA*-infected-untreated mice was significantly upregulated compared to that in the control group (*p* < 0.05). The gene expression of TNF-α in the group treated with Prabchompoothaweep remedy and the Art-PT combination group was significantly downregulated compared to that in the *PbA*-infected-untreated group (*p* < 0.05). In addition, the gene expression of IL-1β and IL-6 in all treated and artesunate groups was significantly lower than that in the *PbA*-infected-untreated group (*p* < 0.05).

Consistent with a previous study in humans, the gene expression of IL-10 was highly upregulated in the *PbA*-infected-untreated group compared to that in the control group (*p* < 0.05). The gene expression of IL-10 was upregulated in mice treated with artesunate or *A. lancea*, Prabchompoothaweep remedy, and Art-PT combination compared to that in the control group (*p* < 0.05), and the gene expression of IL-10 was significantly upregulated in the mice that received the Art-AL combination compared to that in the control group (*p* < 0.05). However, the gene expression of IL-10 in all treated and artesunate groups was also significantly downregulated compared to that in the *PbA*-infected-untreated group (*p* < *0.05*) (Fig. 2D).

The gene expression of CXCL4 (Fig. 2E) and CXCL10 (Fig. 2F) was significantly downregulated in mice that received artesunate, *A. lancea*, Prabchompoothaweep remedy, Art-AL combination, and Art-PT combination groups. In contrast, the gene expression of CXCL4 and CXCL10 was significantly upregulated in the *PbA*-infected- untreated group compared to that in the control group (*p* < 0.05).

The gene expression of VCAM-1 was downregulated in the *A. lacea* treatment and Art-AL combination groups and significantly lower in the Prabchompoothaweep remedy treatment and Art-PT combination groups than those in the *PbA*-infected-untreated group (*p* < 0.05) (Fig. 2G). Moreover, the expression of ICAM-1 (Fig. 2H) and CD36 (Fig. 2I) was significantly reduced in the brains of mice receiving artesunate, *A. lancea*, Prabchompoothaweep remedy, Art-AL combination, and Art-PT combination treatment compared to that in the *PbA*-infected-untreated group (*p* < 0.05).

### Oxidative stress in the brain tissues

**Fig. 3:**
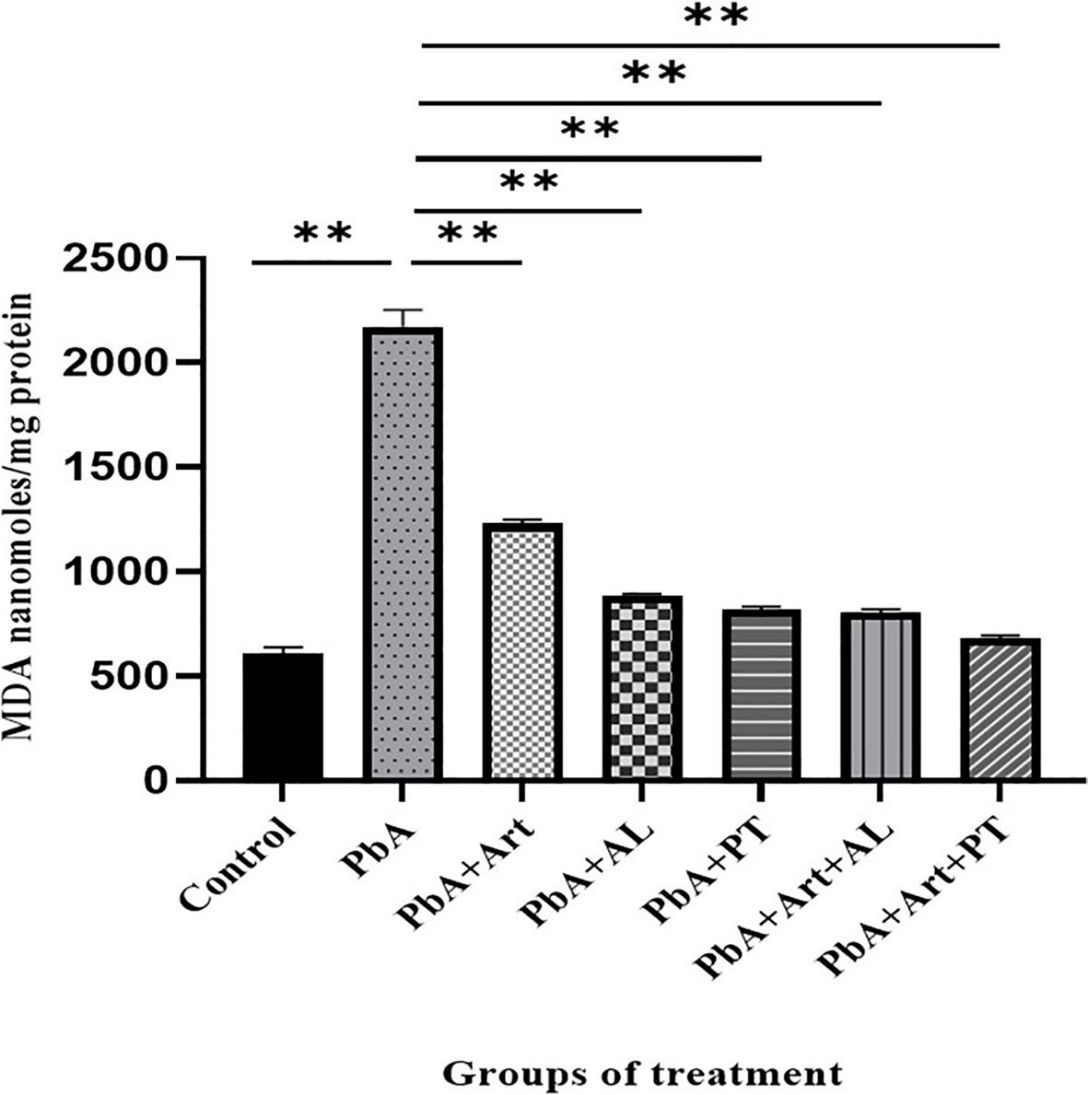
Effect of selected crude extracts from Prabchompoothaweep remedy on oxidative stress in the brain tissues following *PbA-*infected mice by measuring the malondialdehyde (MDA) levels. Brain tissues are harvested at day 13 post-infection. MDA levels are represented as nanomoles/mg protein. *PbA, Plasmodium berghei* ANKA; Art, artesunate; AL, *A. lancea*; PT, Prabchompoothaweep remedy. Results are shown as mean ± standard error of the mean (SEM) (*n* = 5 per group) and are representative of at least two independent experiments. * *p* < 0.05 versus *PbA*, ** *p* < 0.001 versus *PbA*

On day 13 post-infection, MDA levels in the brain homogenates of *PbA*-infected mice were significantly higher than those in the control group (Fig. 3). MDA levels in the brain homogenate of *PbA*-infected mice that received an ethanolic extract of *A. lancea* or Prabchompoothaweep remedy were significantly decreased compared with those in the *PbA*-infected-untreated group (*p* < *0.05*). Moreover, MDA levels in the brain homogenate of *PbA*-infected mice that received adjective therapies (Art-AL combination or Art-PT combinations) were significantly decreased compared with those in the *PbA*-infected-untreated group (*p* < *0.05*).

### Blood–Brain Barrier (BBB) integrity preservation in the brain tissues

**Fig. 4:**
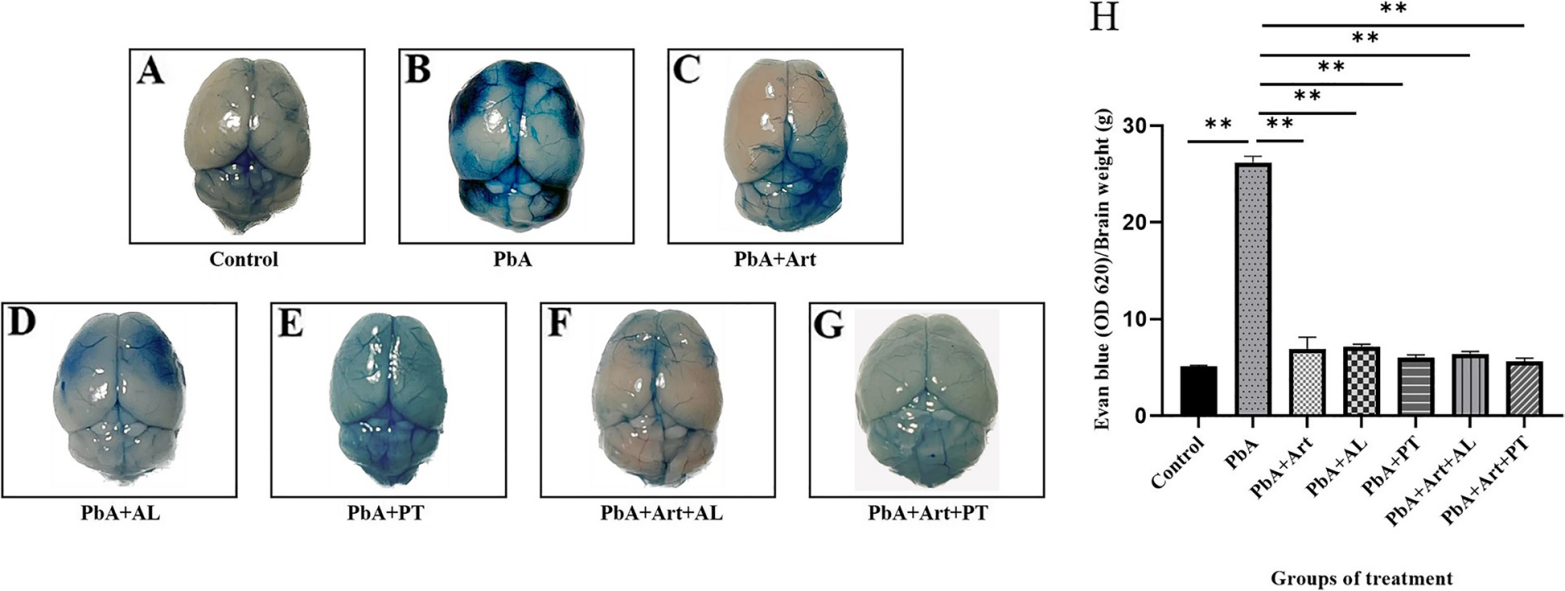
Selected crude extracts from Prabchompoothaweep remedy preserves blood–brain barrier integrity in experimental cerebral malaria (ECM). C57BL/6 were inoculated with *PbA* erythrocytes and treated with selected crude extracts from Prabchompoothaweep remedy for 7 consecutive days. After 13 days post-infection, a solution of 2% Evan blue dye was transcardially injected for approximately 5 min and perfused with saline. Next, the brain was collected, weighed, and photographed. Macroscopic image of the whole brain demonstrating Evan blue dye leakage in cerebral tissue in study groups: (**A**) control, (**B**) *PbA-*infected group, (**C**) artesunate treatment group, (**D**) *A. lancea* treatment group, (**E**) Prabchompoothaweep remedy treatment group, (**F**) Art-AL combination treatment group, and (**G**) Art-PT combination treatment group. (**H**) Quantification of Evan blue dye leakage into the brain at 13 days post-infection. *PbA, Plasmodium berghei* ANKA; Art, artesunate; AL, *A. lancea*; PT, Prabchompoothaweep remedy. Results are shown as mean ± standard error of the mean (SEM) (*n* = 5 per group) and are representative of at least two independent experiments. * *p* < 0.05 versus *PbA*, ** *p* < 0.001 versus *PbA*

The integrity of the BBB was measured using the Evans blue dye assay, as shown in Fig. 4. The results indicated that the control group exhibited no disruption of BBB integrity (Fig. 4A). In contrast, *PbA*-infected-untreated group showed marked accumulation of Evans blue dye in the brain tissue, indicating BBB dysfunction (Fig. 4B). The brain tissue of mice that received artesunate (Fig. 4C), ethanolic crude extracts of *A. lancea* (Fig. 4D), Prabchompoothaweep remedy (Fig. 4E), Art-AL combination (Fig. 4F), and Art-PT combination treatment (Fig. 4G) displayed less staining with Evans blue dye compared with that of the *PbA*-infected-untreated group (Fig. 4B). The brain tissue of *PbA*-infected-untreated mice showed an obvious increase in BBB breakdown (from 5 µg/g in control mice to 26.22 µg/g in *PbA*-infected-untreated mice) (Fig. 4H). In contrast, the brain tissues of mice that received an ethanolic extract of *A. lancea* (7.16 µg/g) or Prabchompoothaweep remedy (6.00 µg/g) had decreased a mean number of Evan blue extravasation into brain parenchyma compared to that in the *PbA*-infected-untreated group. For the adjunctive therapy, brain tissues of mice that received Art-AL (6.41 µg/g) or Art-PT (5.64 µg/g) combinations exhibited a remarkably decreased a mean number of Evan blue extravasation into brain parenchyma compared to that in the *PbA-*infected-untreated group.

### Histological alterations in the brain tissues

**Fig. 5:**
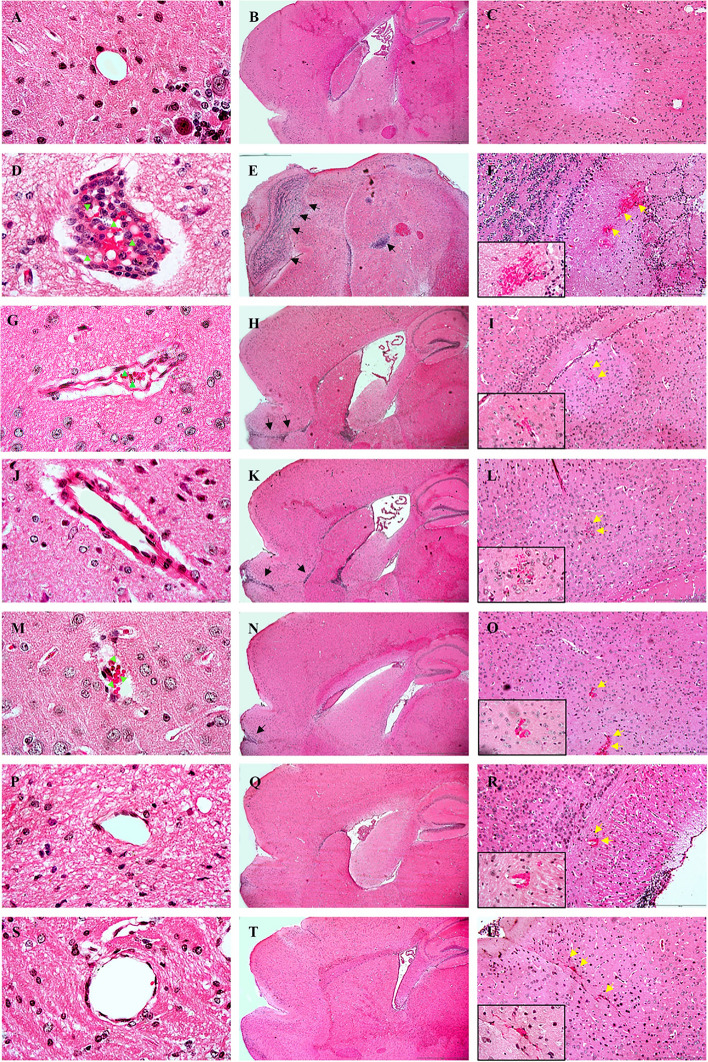
Selected crude extracts from Prabchompoothaweep remedy reduce pathohistological changes in the brain tissue in experimental cerebral malaria (ECM). C57BL/6 were inoculated with *PbA* erythrocytes and treated with selected crude extracts from Prabchompoothaweep remedy for 7 consecutive days. Brain histological sections of the control (**A**, **B**, **C**), infected untreated (**D**, **E**, **F**), artesunate treatment (**G**, **H**, **I**), *A. lancea* treatment (**J**, **K**, **L**), Prabchompoothaweep remedy treatment (**M**, **N**, **O**), Art-AL combination (**P**, **Q**, **R**), and Art-PT combination groups (**S**, **T**, **U**) stained with H&E solution*.* The control group shows the normal histological aspect in the cerebral cortex, containing healthy neurons and normal vessels (**A**), no leukocyte infiltration (**B**), and no brain hemorrhage (**C**). Arrowhead indicates a large number of infected red blood cells within the brain microvasculature of the infected group (**D**), while a small number of infected red blood cells are observed in the Prabchompoothaweep remedy group (**M**). The black arrow shows a large area of leukocyte infiltration in the infected group (**E**), whereas a small area of infiltration is observed in Art (**H**), *A. lancea* (**K**), and Prabchompoothaweep remedy (**N**). Yellow arrow demonstrates an extensive hemorrhagic area in the infected group (**F**), while a small hemorrhagic area is detected in the brain of mice treated with Art (**I**), *A. lancea* (**L**), Prabchompoothaweep remedy (**O**), Art-AL combination (**R**), and Art-PT combination group (**U**). Art, artesunate; AL, *A. lancea*; PT, Prabchompoothaweep remedy. The magnifications are 4X for panels B, E, H, K, N, Q, and T, 20X for panels C, F, I, L, O, R, U, and 100X for panels A, D, G, J, M, P, S

At 13 days post-infection, brain sections of the control group did not show alterations in the cellular parenchyma, such as vascular dilation, capillary obstruction (Fig. 5A), leukocyte accumulation (Fig. 5B), or brain hemorrhage (Fig. 5C). Meanwhile, the brain sections of the *PbA*-infected-untreated group displayed prominent intravascular accumulation of parasitized red blood cells and leukocytes in the brain vessels (Fig. 5D). In addition, inflammatory cell infiltrates (Fig. 5E) and several hemorrhagic areas (Fig. 5F) were detected in the cerebral parenchyma of the *PbA*-infected-untreated group. In contrast, all histopathological alterations were reduced in *PbA*-infected mice that received artesunate, *A. lancea* or Prabchompoothaweep remedy. In cortical brain sections with severe inflammation, mice in a group of artesunate (Fig. 5G, H, I), *A. lancea* (Fig. 5J, K, L) and Prabchompoothaweep remedy (Fig. 5M, N, O) exhibited a less diffuse cellular infiltration, hemorrhage area, and occluded capillaries compared to the brains of the *PbA*-infected-untreated group. Moreover, brain sections of *PbA*-infected mice that received the Art-AL (Fig. 5P, Q, R) or Art-PT combination (Fig. 5S, T, U) showed mild lesions, with lower numbers of parasitized red blood cells and leukocytes obstructing brain vessels than the *PbA*-infected-untreated group.

### Long-term memory impairment

**Fig. 6:**
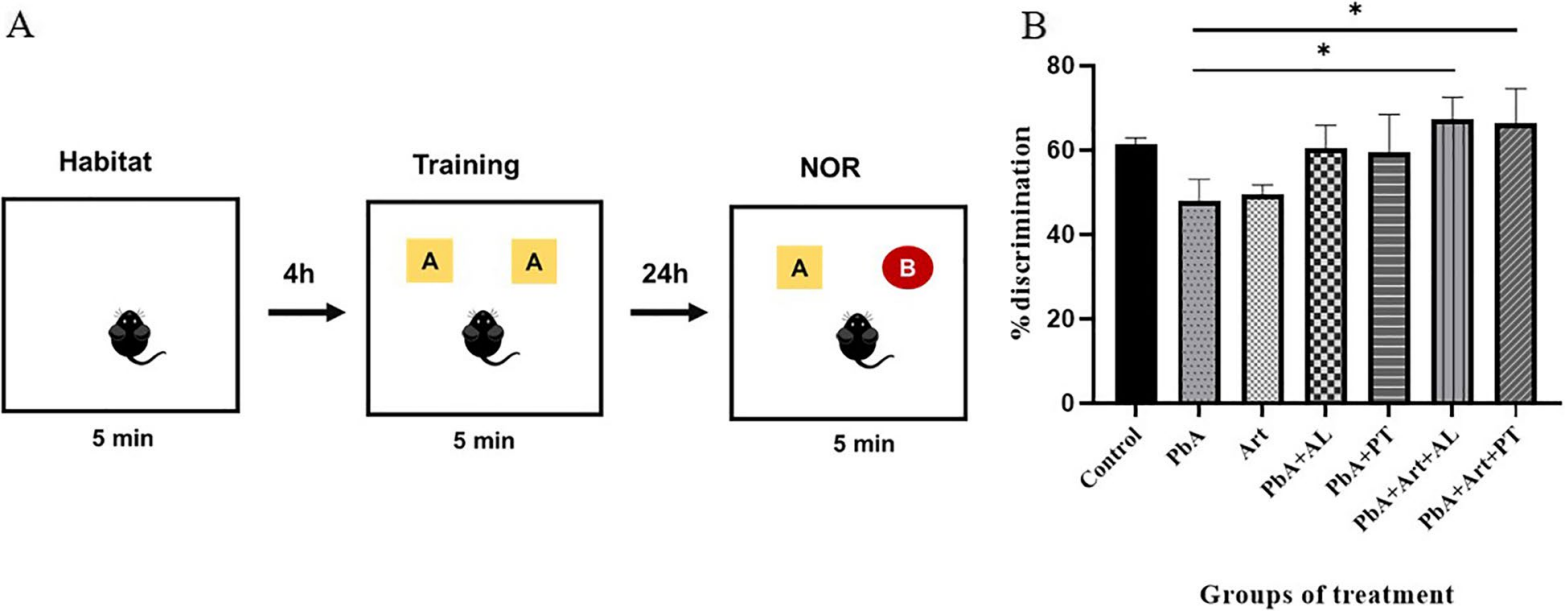
Effect of selected crude extracts from Prabchompoothaweep remedy on long-term memory impairment following *PbA-*infected mice using novel object recognition test. Mice are submitted to a novel object recognition test on day 12 post-infection for habituation and training session and day 13 post-infection for the testing session. **A** Illustration of novel object recognition test protocol. **B** The percentage of discrimination index among the groups. *PbA, Plasmodium berghei* ANKA; Art, artesunate; AL, *A. lancea*; PT, Prabchompoothaweep remedy: NOR: novel object recognition. Results are shown as mean ± standard error of the mean (SEM) (*n* = 5 per group) and are representative of at least two independent experiments. * *p* < 0.05 versus *PbA*, ** *p* < 0.001 versus *PbA*

On day 13 post-infection, the *PbA*-infected-untreated group presented impairment in novel object recognition memory, with a lower percentage of discrimination than the control group (*p* < 0.05). In contrast, *PbA*-infected mice administered the ethanolic extract of *A. lancea* or Prabchompoothaweep remedy demonstrated an increase in recognition memory compared to the *PbA*-infected-untreated group. Furthermore, *PbA*-infected mice administered Art-AL and Art-PT combinations had a significantly increased discrimination index compared with the *PbA*-infected-untreated group (*p* < 0.05) (Fig. 6B).

## Discussion

Malaria is an infectious disease that generally affects children and adults living in tropical countries, and several studies have reported its deleterious effects on the central nervous system. Cognitive dysfunction is a common neurobehavioral sequela in survivors [2]. The pathogenesis of CM in humans is characterized by parasitized sequestration in the microcapillaries, which leads to BBB dysfunction. The most prominent pathogenesis in humans is similar to ECM characteristics, such as hyperinflammation, intravascular accumulation of immune T cells, an increase in oxidative stress, and BBB breakdown [44]. Although the use of artesunate, a fast-killing parasite, offers significant improvement in CM management, the remaining majority of deaths and neurological impairments require research on adjunctive therapies [37]. Additionally, pharmacological or nutritional interventions that attempt to abolish oxidative stress and inflammation in conventional treatments may improve therapeutic outcomes in CM as depicted through a recent study [45]. Natural products are generally a source of compounds with important and valuable pharmacotherapeutic properties, and a diet rich in these products is known to have numerous benefits for human health [46]. Moreover, natural products contain various phytochemical compounds that can modulate different signaling pathways involved in various pathological conditions, such as cancer, vasculopathy, and neurodegenerative disorders (Parkinson’s and Alzheimer’s) [47] as well as infectious diseases, such as leishmaniasis and malaria [48, 49]. Therefore, the present study highlights the use of natural products to trigger the development of novel effective anti-malarial drugs with neuroprotective effects. In our previous study, the ethanolic extracts of the rhizome of *A. lancea* and Prabchompoothaweep remedy had a strong in vitro anti-plasmodium activity against the *P. falciparum* K1 strain (IC_50_ values of 7.37, and 14.13 µg/mL) and also presented low toxicity to the Vero cell line (a selectivity index higher than 2 indicates nontoxicity of the compound) [24]. Furthermore, high parasitemia suppression was observed in both *A. lancea* at 400 mg/kg (60.09%) and Prabchompoothaweep remedy at 600 mg/kg (60.10%). The LD_50_ values of these extracts exceeded 2,000 mg/kg, which is safe in an acute oral toxicity test in a mouse model [25]. Furthermore, *A. lancea* has various therapeutic properties, including anti-cancer, anti-inflammation, and anti-microbial activities, as well as effects on various organs, including the central nervous, cardiovascular, and intestinal systems [50]. The pharmacological properties of Prabchompoothaweep remedy, including antiallergic activity, anti-inflammation, and anti-oxidant properties, have been reported [23]. *A. lancea* and Prabchompoothaweep remedy were investigated for ECM in this study because their ethanolic extracts demonstrated promising antimalarial activity in vitro and in vivo with low toxic effects.

In the present study, selected crude extracts from the Prabchompoothaweep remedy exhibited the suppression of parasitemia, which showed a high percentage in the infected mice that received Art-PT combination (82.27%), followed by those that received Art-AL combination treatment (78.27%), those that received *A. lancea* (54.41%), and those that received Prabchompoothaweep remedy (41.13%). In this context, artesunate demonstrated a direct killing effect on *Plasmodium* parasites [51]. Moreover, our findings are in agreement with previous studies that demonstrated the activity of natural plant extracts, such as *Zizyphus spinachristi* and *Terminalia albida*, in reducing parasitemia in murine CM [34, 52]. These crude extracts inhibited schizonticide activity in *PbA-*infected mice. Moreover, antimalarial activities might be related to secondary bioactive compounds, such as flavonoids, polyphenols, alkaloids, terpenoids, and saponins [53]. Our previous phytochemical screening revealed that the ethanolic extracts of *A. lancea* and Prabchopoothaweep remedy contain several plant secondary metabolites. *A. lancea* consists of terpenoids, alkaloids, and coumarins, while Prabchompoothaweep remedy consists of terpenoids, alkaloids, tannins, and coumarins [25]. These results are in agreement with those of a previous study on secondary metabolites, which suggested that alkaloids, cyclic terpenes, flavonoids, xanthones, anthraquinones, phenols, sesquiterpenes, and other compounds show antimalarial activity [54]. Among these compounds, terpenoids play a role in endoperoxidation, which produces potentially toxic heme-adducts in the parasite. Alkaloids (quinine) may exert antimalarial activities by controlling protein synthesis and arresting toxic heme converted to non-toxic hemozoin pigments in the food vacuole of the parasite [55]. Next, tannins act as antimalarial agents by neutralizing reactive oxygen species. Additionally, coumarin compounds can regulate superoxide dismutase (oxidative enzymes) and downregulate DNA synthesis in parasites. The anti-oxidant effects can prevent heme polymerization, which is toxic to intra-erythrocytic parasites [15]. Pyrogallol, a phenol compound, possessed the antimalarial effect by inhibiting the proton pump, V-type H ^+^ -ATPase and causing the digestive vacuole pH alteration [56]. From our previous LC–MS analysis, we found that *A. lancea* is the main source of secondary compounds, including chlorogenic acid, 1,2,6,8-tetrahydroxy-3-methyl anthraquinone 2-O-b-D-glucoside, taraxacolide 1-O-b-D-glucopyranoside, and salicylic acid. Analysis of Prabchompoothaweep remedy extracts revealed the presence of luteolin, 6’-O-formylmarmin, caffeic acid, eudesmic acid, gallic acid, and ellagic acid constituents [25]. Among these compounds, caffeic acid has plenty of pharmacological effects, including antioxidant activity, anti-inflammation, antineoplastic properties, and antimalarial activity [57]. Caffeic acid and its derivatives showed a growth inhibition of 55% at 100 mg/kg in 4-day suppressive test [58]. Anti-inflammation, anti-allergy, anti-cancer, and antioxidant activity have been reported in luteolin [59]. Gallic acid is known to have anti-cancer, antibacterial, and anti-plasmodium activities [60]. Ellagic acid, isolated from *Terminalia mollins,* has strong anti-malarial activity (IC_50_ = 0.175 µg/mL) [61]. Furthermore, Soh et al. reported that ellagic acid suppresses parasitemia in a dose-dependent manner with 100% suppression in mice treated with 50 and 100 mg/kg via the intraperitoneal route [62]. Previous studies supported the idea that ellagic acid altered the pH of the digestive vacuole through the prevention of proton pumps that control the acidification of this organelle [63]. In conclusion, the ethanolic extracts of *A. lancea* and Prabchopoothaweep remedy monotherapy inhibit parasitemia progression, whereas a combination of artesunate (standard drug) and crude extracts from Prabchompoothaweep remedy significantly exerts a potent synergistic effect in the antimalarial property. This observation demonstrated the possible synergistic effect between the combination of artesunate and crude extracts from Prabchompoothaweep remedy, as artesunate is presented to be rapid absorbed in the tissues and quickly excreted after administration. Furthermore, previous study suggested that the crude extracts might aim to enhance the bioactivity of artesunate and the permeability of the drug in infected red blood cells [20]. This leads to the rapid clearance of parasites with no recrudescence in the early phase of infection, whereas a slow clearance of parasites with recrudescence was observed in monotherapy. So, the rapid and almost complete parasite clearance might prevent parasite sequestration into the brain, contributing to reduce pathogenesis of cerebral malaria.

Cellular and histological changes, including parasite sequestration, brain inflammation, and BBB breakdown, are associated with CM pathogenesis, which can contribute to major behavioral impairment in infected animals [64]. ECM, a murine model of cerebral malaria, is an available tool for developing effective therapies, provided that mice reproduce most of the characteristics and symptoms observed in humans [65]. In this study, *PbA*-infected mice demonstrated signs of human CM, such as muscle weakness, abnormal gait pattern, poor reflexes, roll, and convulsions on days 5–6 after infection. In contrast, mice treated with *A. lancea* and Prabchompoothaweep remedy presented a significant increase in the RMCBS score with mild evidence of neurological impairment. Additionally, mice treated with Art-AL combination treatment and Art-PT combination treatment showed a progressive significant improvement in survival rate, remaining alive until 13-days post-infection without evidence of neurological impairments. This study was consistent with a previous study that suggested that an increase in survival rate is usually related to neurological parameter improvement [66, 67]. This could be related to the presence of bioactive compounds in the crude extracts, such as luteolin [59], vanillic acid [68], kaempferol [69], caffeic acid [57], gallic acid [60], and ellagic acid [70]. These compounds have been shown to possess anti-inflammation and anti-oxidant activities. Therefore, it may reduce the pathogenesis of the disease and improve survival and cerebral symptoms in the ECM [71].

Based on this evidence, BBB disruption is caused by a two-consequence process after infection with *Plasmodium*. First, parasitized erythrocytes can obstruct brain vessels, triggering local hypoxia. Secondly, the parasitized erythrocytes can evoke an inflammatory cytokine response in the brain, contributing to excessive pro-inflammatory cytokines and reactive oxygen species [72]. This study showed that *PbA-*infected mice exhibited an increase in TNF-α, IL-1β, and IL-6 expression levels in the brain at day 13 post-infection. In contrast, the infected mice that received *A. lancea* and Prabchopoothaweep remedy alone could lead to a significant reduction in TNF-α, IL-1β, and IL-6 expression in the brain of *PbA-* infected mice, while their combination results further significantly downregulated TNF-α, IL-1β, and IL-6 expression, suggesting a synergistic effect of Art-AL and Art-PT combinations. IL-10, an immune-regulatory cytokine, plays a protective role in ECM development [73]. In this study, the expression of IL-10 increased in the *PbA-*infected-untreated group. Although the ethanolic crude extracts of *A. lancea*, Prabchopoothaweep remedy, and combination treatment extended the survival of ECM-susceptible mice, all treatments downregulated the expression of IL-10 in the *PbA-*infected-untreated group. These results are consistent with those of Crowley et al., who showed that the expression of IL-10 was reduced in the ECM after treatment with synthetic oleanane triterpenoids [18]. Although high levels of the anti-inflammatory cytokine IL-10 could have a potential role in human malaria by preventing parasite-triggered pro-inflammatory responses that lead to the severity of disease progression, many reports have suggested that high levels of IL-10 might also disturb parasite elimination [74]. Additionally, inflammatory cytokines are related to brain volume and directly affect endothelial cells and their receptors [75]. In this case, *A. lancea* and Prabchopoothaweep remedy alone downregulated the production of ICAM-1, VCAM-1, and CD36 on day 13 post-infection, while the expression of ICAM-1, VCAM-1, and CD36 was significantly lower than that in the Art-AL and Art-PT combination treatment groups. CXCL4 and CXCL10 chemokines participate in the pro-inflammatory response and are usually used to predict the severity of ECM [76]. The results of the present study showed that the *A. lancea* and Prabchopoothaweep remedy significantly downregulated the expression of CXCL4 and CXCL10 in brain tissues of the *PbA-*infected-untreated group, while the combination of Art-AL and Art-PT treatment further significantly downregulated the expression of these two markers. These results are in accordance with those of Lyke et al. [77] and Wunderlich et al. [78] who demonstrated that children and mice that developed severe cerebral malaria showed a high level of inflammatory response. Thus, the reduction in the levels of cytokines, chemokines, and adhesion molecules in treated mice could be related to brain protection and an increase in survival, as observed in this study. Moreover, several studies have demonstrated that the chemical composition of *A. lancea* and Prabchompoothaweep remedy confers it valuable anti-inflammation property. It can support from previous works, which found that treatment with *A. lancea* and Prabchompoothaweep remedy reduce the level of pro-inflammatory cytokines, such as TNF-α and IL-6 [29]. Altogether, this study showed that the combination of Art-AL and Art-PT acted synergistically to reduce the immune response and the accompanying levels of chemokines and adhesion molecules, although the degree of effect varied among the factors.

Oxidative stress in brain endothelial cells has been linked to ECM development. Based on this evidence, amino acids from hemoglobin are important for the development of parasites during the erythrocytic stage of the malaria parasite. Therefore, the appearance of parasites facilitates the degradation of the host hemoglobin. The extent of hemoglobin degradation is associated with malaria severity. The lowered level of hemoglobin would indicate increased oxidant stress, identified by an increase in MDA levels [79]. The present study demonstrated that *A. lancea*, Prabchopoothaweep remedy treatment alone, and Art-AL combination treatment significantly lowered the production of MDA on day 13 post-infection, while the production of MDA was significantly lower in Art-PT combination than that in *PbA*-infected mice. It is well known that the beneficial effects of natural products is related to several bioactive molecules; however, their anti-oxidant effect could be related to phenolic components, such as orientin, homoorientin, catechin, epicatechin, ferulic acid, vanillic acid, gallic acid, p-hydroxybenzoic acid, and syringic acid [80]. This is in accordance with our previous study on liquid chromatography–mass spectrometry analysis. *A. lancea* and Prabchopoothaweep remedy also contained several phenolic compounds. Phenolic compounds are excellent anti-oxidant owing to the presence of a hydroxyl group, which is available to capture oxygen free radicals. They can rebalance the production of oxygen species, which reduces cell damage caused by free radicals [67]. These results suggest that treatment with *A. lancea*, Prabchompoothaweep remedy, Art-AL, and Art-PT can provide anti-oxidant activity to the brain in the ECM. Additional studies are required to investigate the precise molecular component of *A. lancea* and Prabchompoothaweep remedy that is responsible for anti-oxidant activity leading to the protection observed in ECM.

An increase in inflammatory levels leads to vascular leakage and may result in microcirculatory dysfunction and neurological impairment. The activation of death pathways in endothelial cells directly triggers T lymphocyte damage to BBB integrity. The persistence of infection resolution following antimalarial treatment might be related to ECM outcomes. To mimic these events with great clinical relevance, patients tend to receive treatment late in the course of the disease; therefore, in this study, mice were treated after the onset of neurological symptoms with the ethanolic crude extract of *A. lancea*, Prabchompoothaweep remedy, Art-AL combination, and Art-PT combination. The results showed that all treatments prevented Evans blue dye extravasation into brain tissue. These treatments improved the survival rate and prevented neurological impairment in mice, suggesting that the standard drug and crude extracts might not only protect the BBB but also maintain its function and lessen the symptoms associated with neurological injury. Rapid reversibility of cerebral edema has also been observed in adults and children following antimalarial therapy, highlighting the need for novel malaria therapies that can prevent and reverse the cytotoxicity of neurons [81]. These findings agree with those of a previous study; the components in plants (triterpenoids and synthetic oleanane triterpenoids) showed neuroprotective effects in preclinical models of Huntington’s disease-induced neurotoxicity [18]. The mechanism by which crude extracts prevent BBB breakdown may be related to the modulation of adhesion proteins in brain endothelial cells. Therefore, additional studies are required to confirm this hypothesis. Moreover, a previous report showed a strong connection between BBB breakdown and CM pathogenesis in both human and mouse models [82]. The present study found that parasitemia levels were relatively low in *A. lancea*, Prabchompoothaweep remedy, and combination treatment administered to *PbA*-infected mice, and there was a significant reduction in brain edema after receiving crude extracts and the combination treatment on day 6 post-infection. *A. lancea* and Prabchompoothaweep remedy also reduced brain histopathological alterations induced by *PbA* infection. Furthermore, the combination treatment significantly attenuated disarrangement in the brain cortex with healthy neurons, normal vessels, no leukocyte infiltration, and a few areas of brain hemorrhage in *PbA*-infected mice. These results provide evidence that the combination treatment has the potential to reduce brain neuroinflammation associated with ECM pathogenesis.

Behavioral alteration in the central nervous system is commonly associated with BBB dysfunction. Several studies have demonstrated that *P. falciparum* infection causes and triggers long-term neurological impairments, such as memory deficits. An event associated with a deficit has been reported to be related to inflammatory and oxidative stress [3]. This immune activation during disease progression or stress conditions might suppress neurotrophic factors, which play important roles in modulating neuronal survival and plasticity mechanisms in learning and memory, as a result of lessening the effects on adult neurogenesis and cognitive function [83]. Moreover, several studies have shown cognitive-behavior alterations in experimental models of malaria [84]. We also observed an increase in long-term memory impairment in *PbA*-infected mice, while the ethanolic extract of *A. lancea* and Prabchompoothaweep remedy mitigates behavioral alteration. Additionally, mice treated with Art-AL and Art-PT combination treatment showed a progressive significant improvement in cognitive impairment when compared with the *PbA-*infected group. These results are in line with a previous study that demonstrated that the co-administration of standard drugs and adjunctive therapy can improve cognitive impairment [84]. BBB breakdown has been related to pro-inflammatory influx into brain tissue and consequent behavioral alterations. *A. lancea* and Prabchopoothaweep remedy exert anti-inflammatory and anti-oxidative effects. They can inhibit oxidative stress, maintain anti-oxidant enzyme activities, and reduce cytokine levels. The protective effects on BBB by selected crude extracts from Prabchompoothaweep remedy might result in a reduction in microglia and astrocytes activation, which can prevent neuroinflammation and the development of neurological damage. Further research is required to explore the mechanisms of improvement in cognitive impairment after treatment with the Art-AL and Art-PT combinations.

At length, this first complete set of studies demonstrated a large number of anti-plasmodium efficacy of selected crude extracts with plausible synergistic activity with artesunate against *P. berghei* induced ECM. These combined treatments contribute to the rapid clearance of parasites with no recrudescence in the early phase of infection. In the case of combination treatment, significantly higher (80%) chemosuppression was observed as compared to individual dosing of the combination partner (50%). Moreover, the nearly complete parasite clearance might prevent parasite sequestration into the brain and reduce the pathological processes caused by malaria, such as inflammation, oxidative stress, and microvascular dysfunction. Based on this result, the combination treatment is in consonance with the regulated interplay of inflammatory cytokines, chemokines, endothelial activation markers, and antioxidant properties after treatment, resulting in the recovery of clinical outcome and/or decrease in mortality rate, together with the improvement of long-term neurocognitive impairment.

## Conclusions

In the present study, we demonstrated that the oral co-administration of Art and AL and the co-administration of Art and PT exhibited antimalarial activity, anti-inflammatory and anti-oxidative properties in *PbA* infection. Co-administration also preserved BBB integrity and improved neurocognitive deficits, which inhibited the development of cerebral malaria. Moreover, co-administration is effective in protecting against ECM development and might represent an alternative therapeutic approach for the treatment of cerebral malaria. However, further studies are needed to identify the active compounds isolated from *A. lancea* and Prabchompoothavep responsible for their anti-malarial properties and to establish their molecular mechanisms of action. Further investigations of the prophylactic and long-term effects on the outcome of cerebral malaria are recommended.

## Data Availability

The data associated with this study have been included in this published article. Additional files are available from the corresponding authors upon request.

## Abbreviations

BBB: Blood–brain barrier
MDA: Malondialdehyde
ECM: Extracerebral malaria
